# Improved phylogenetic resolution within the Neotropical rainforest genus *Zygia* (Mimoseae, Fabaceae) using phylogenomic data

**DOI:** 10.3389/fpls.2026.1816329

**Published:** 2026-06-12

**Authors:** Julia Ferm, R. Toby Pennington, Gwilym P. Lewis, Flávia F. Pezzini, Rowan J. Schley, Olle Thureborn, Audrey Farbos, Catarina Rydin

**Affiliations:** 1 Department of Ecology, Environment and Plant Sciences, Stockholm University, Stockholm, Sweden; 2 Royal Botanic Garden Edinburgh, Edinburgh, United Kingdom; 3 Department of Geography, University of Exeter, Exeter, United Kingdom; 4 Department of Biology, Washington University, St Louis, MO, United States; 5 Missouri Botanical Garden, St Louis, MO, United States; 6 Department of Accelerated Taxonomy, Royal Botanic Gardens Kew, London, United Kingdom; 7 Bergius Botanic Garden, Stockholm University, Stockholm, Sweden; 8 University of Exeter Sequencing Facility, Exeter, United Kingdom

**Keywords:** Mimoseae, neotropical, phylogenomics, phylogeny, *Zygia*

## Abstract

**Introduction:**

The Neotropical legume genus *Zygia* consists of approximately 60 species of small, cauliflorous trees. Phylogenetic relationships within *Zygia* are not fully known since previous phylogenies were for the most part poorly supported and unresolved with non-monophyletic species. Previous studies have shown that the genus *Marmaroxylon* is nested within *Zygia*, but a full sample of *Marmaroxylon* has yet to be included in analyses. This study aims to resolve taxonomic limits of *Zygia* and to test if phylogenomic data and more sampling of taxa improve resolution within the genus.

**Methods:**

We utilize data from 1,315 nuclear loci sequenced from 134 accessions, representing 47 species of *Zygia*, as well as four species of the closely related genus *Macrosamanea*, the poorly known *Marmaroxylon magdalenae*, endemic to the Magdalena Valley in Colombia, and *Marmaroxylon eperuetorum*, which is the last remaining species of *Marmaroxylon* not tested with DNA data. The data are analyzed using species tree reconstruction based on the multispecies coalescent model and maximum likelihood analyses of concatenated datasets.

**Results:**

Our phylogenetic analysis showed that the core *Zygia* clade is sister to a clade consisting of *Macrosamanea*, *Zygia turneri*, *M. magdalenae*, *Inga*, *Ingopsis*, and *Pseudocojoba*. *Zygia* is non-monophyletic since *Z. turneri* is more closely related to *M. magdalenae* and *Macrosamanea* than to the remaining species of *Zygia*. *Marmaroxylon eperuetorum* is found within the core *Zygia* clade. Resolution within *Zygia* clearly improved with phylogenomic data and denser sampling of taxa, but non-monophyletic species are still present.

**Discussion:**

The core *Zygia* clade is strongly supported statistically and relationships within it are well-resolved. However, 13 species of *Zygia* are non-monophyletic, and the reasons for this are not yet fully known but could be due to, e.g., misidentification, hybridization, or incomplete lineage sorting. Both species trees reconstructed based on the multispecies coalescent model and phylogenetic trees inferred from maximum likelihood analyses of the concatenated datasets show similar topologies. Gene tree discordance is high for many branches within *Zygia* and is, in most cases, due to incomplete lineage sorting. To render *Zygia* monophyletic, new combinations in *Macrosamanea* are given for *Z*. *turneri* and *Marmaroxylon magdalenae*.

## 1 Introduction

*Zygia* P.Browne (mimosoid clade, Fabaceae) is a genus of approximately 60 rainforest tree species found throughout the Neotropics. Some species are widespread across the Amazon basin, a few with an even wider distribution covering South America, Central America, and the Caribbean. Others have a more restricted distribution, and some species are known only from the type locality (Barneby and Grimes, 1997).

**Figure 1:**
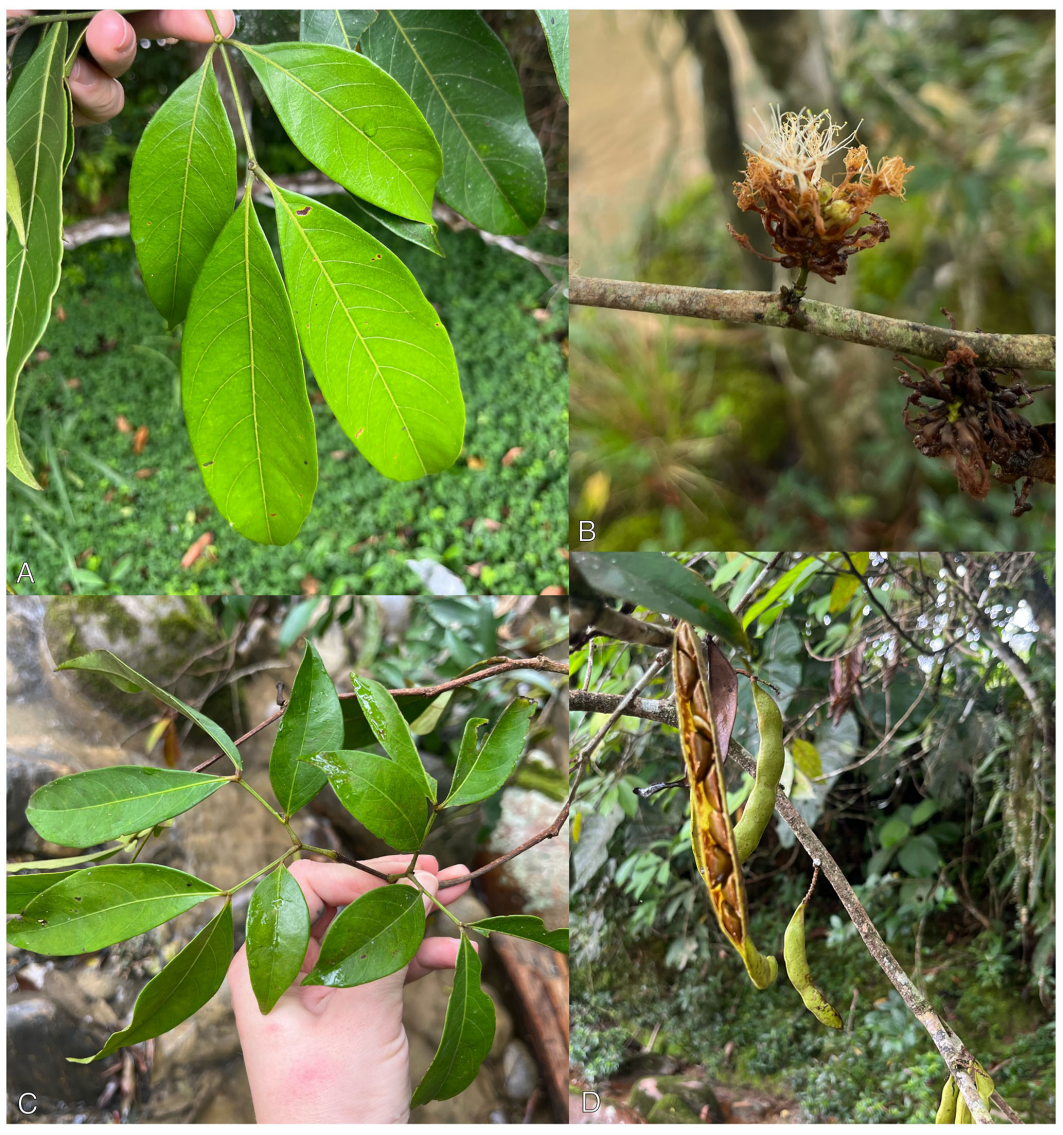
*Zygia longifolia.* **(A)** Part of a bipinnate leaf with extrafloral nectaries between leaflet pairs. **(B)** Cauliflorous inflorescence with mainly post-anthesis flowers and white stamens. **(C)** Bipinnate leaves. **(D)** Dehiscent pods with seeds. Photos: Julia Ferm, Rio Bombuscaro, Zamora, Ecuador 2025.

The latest monograph of *Zygia* recognized 58 species placed in nine sections based on morphology, and included the genus *Marmaroxylon* Killip in synonymy (Barneby and Grimes, 1997). Since then, two new species of *Zygia* have been described (Ståhl et al., 2010; Vargas and Moreno, 2024). *Zygia* species are recognized as small trees or shrubs, rarely up to 20 m in height, with some reports of individuals 35 m tall (e.g., *Z. collina* [Sandwith] Barneby & J.W.Grimes and *Z. tetragona* Barneby & J.W.Grimes). They either have bipinnate leaves with one pair of pinnae and a few large leaflets on each pinna, or have several pairs of pinnae with many smaller leaflets on each pinna. The pods are dehiscent and are straight to decurved and either flattened or cylindrical, and their surface can be smooth or rough. *Zygia* is further characterized by the striking feature of cauliflory; white–green to pink flowers may cover the stems and branches beneath the foliage (**Figures 1**, Rico-Arce, 1991; Rico, 1994; Barneby and Grimes 1997). *Zygia* occurs in wet environments like lowland tropical forests, riparian forests, shore habitats, and mangrove swamps, and in the Andes, a few are found on high altitudes (e.g., *Z*. *lehmannii* [Harms] Britton & Rose) (Barneby and Grimes, 1997).

**Figure 2:**
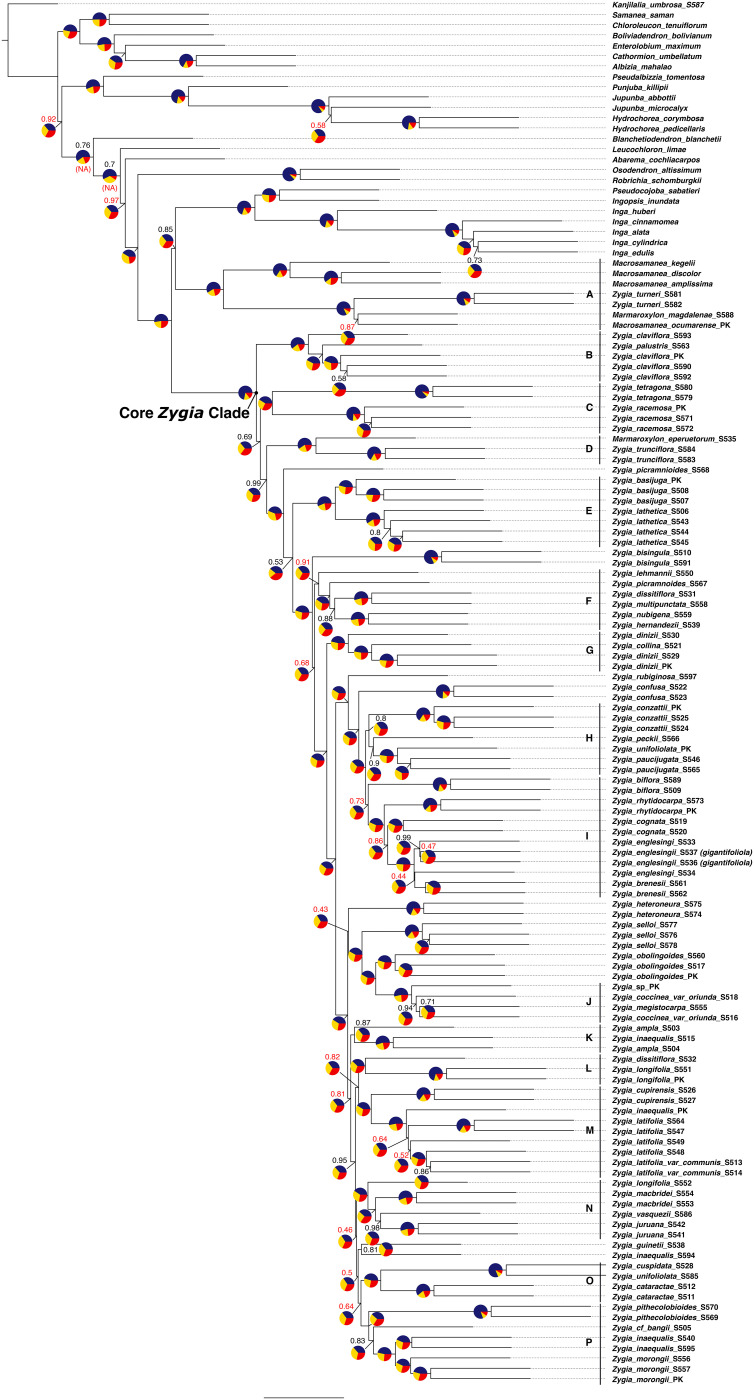
Species tree assembled in Astral-III based on data from all accessions and 1172 loci (i.e. multiple accessions for some species and a total of 134 taxa and gene trees of putatively paralogous loci and loci with ³50% missing data or ³50% missing taxa excluded). Quartet frequencies are presented as pie charts on nodes. Pie charts on branches show relative quartet frequencies for the three quartet topologies: blue = species tree topology; yellow = first alternative topology; red = second alternative topology. Local posterior probability values (LPP) <1 are presented, with values at nodes that got *p*-values ≥ 0.05 from the polytomy test indicated in red.

The first phylogenetic analysis of *Zygia* based on plastid and nuclear DNA data showed that most species of *Marmaroxylon* were nested among species of *Zygia* (Ferm et al., 2019), agreeing with Barneby and Grimes (1997), but none of the sections were recovered. Moreover, *Zygia* was non-monophyletic with a few species resolved outside of a core *Zygia* (and *Marmaroxylon*) clade. This core *Zygia* clade was sister to a clade comprising *Macrosamanea pubiramea* (Steud.) Barneby & J.W.Grimes and *Marmaroxylon ocumarense* (Pittier) L.Rico (Ferm et al., 2019). Relationships within the core *Zygia* clade were for the most part poorly supported and unresolved. Eight species were recovered as non-monophyletic, which, according to Ferm et al. (2019), could be due to cryptic species or indicate the need for more taxonomic studies. Ferm et al. (2019) also mentioned other reasons for non-monophyly in widespread rainforest species as stated by Pennington and Lavin (2016), i.e., huge population sizes and long life spans combined with effective pollen flow and seed dispersal, which will lead to preservation of ancestral genetic polymorphism since it will take a long time for coalescence to occur; in other words, incomplete lineage sorting (ILS). Furthermore, Ferm et al. (2019) revealed that *Zygia inundata* (Ducke) H.C.Lima ex Barneby & J.W.Grimes and *Zygia sabatieri* Barneby & J.W.Grimes were closely related to the genus *Inga* Mill. Following these results, also confirmed by later work (e.g., Ringelberg et al., 2022), *Z. inundata* was placed in the monospecific genus *Ingopsis* (Barneby & J.W.Grimes) Ferm, *Z. sabatieri* was placed in the monospecific genus *Pseudocojoba* (Barneby & J.W.Grimes) Ferm, and *M. ocumarense* was transferred to *Macrosamanea* Britton & Rose (Ferm and Rydin, 2024).

Given the previous taxonomic and phylogenetic uncertainty surrounding species relationships in *Zygia* based on single-locus genetic data, this study aims to untangle phylogenetic relationships in *Zygia* and its close relatives using >1,300 genes sequenced at phylogenomic scale. We also include a more comprehensive sampling of *Zygia* species than used in any previous study. Rare species that have been difficult to obtain leaf material from for DNA sequencing in the past (e.g., *Zygia rhytidocarpa* L.Rico, *Z. rubiginosa* L.Rico & Q.Jiménez, and *Z. vasquezii* L.Rico), as well as the final species of *Marmaroxylon* to be analyzed with DNA sequence data (*M. eperuetorum* [Sandwith] L.Rico) are included here. To ensure robust phylogenetic inference, different ways of handling putatively paralogous genes and missing data were used and the results were compared.

Using the new phylogenetic tree we infer in this study, we aimed to (1) resolve taxonomic limits of *Zygia*, especially in relation to the genera *Marmaroxylon*, *Macrosamanea*, *Inga*, *Ingopsis*, and *Pseudocojoba*, and (2) test if phylogenomic data and expanded sampling of taxa improve resolution of species relationships within *Zygia*.

**Figure 3:**
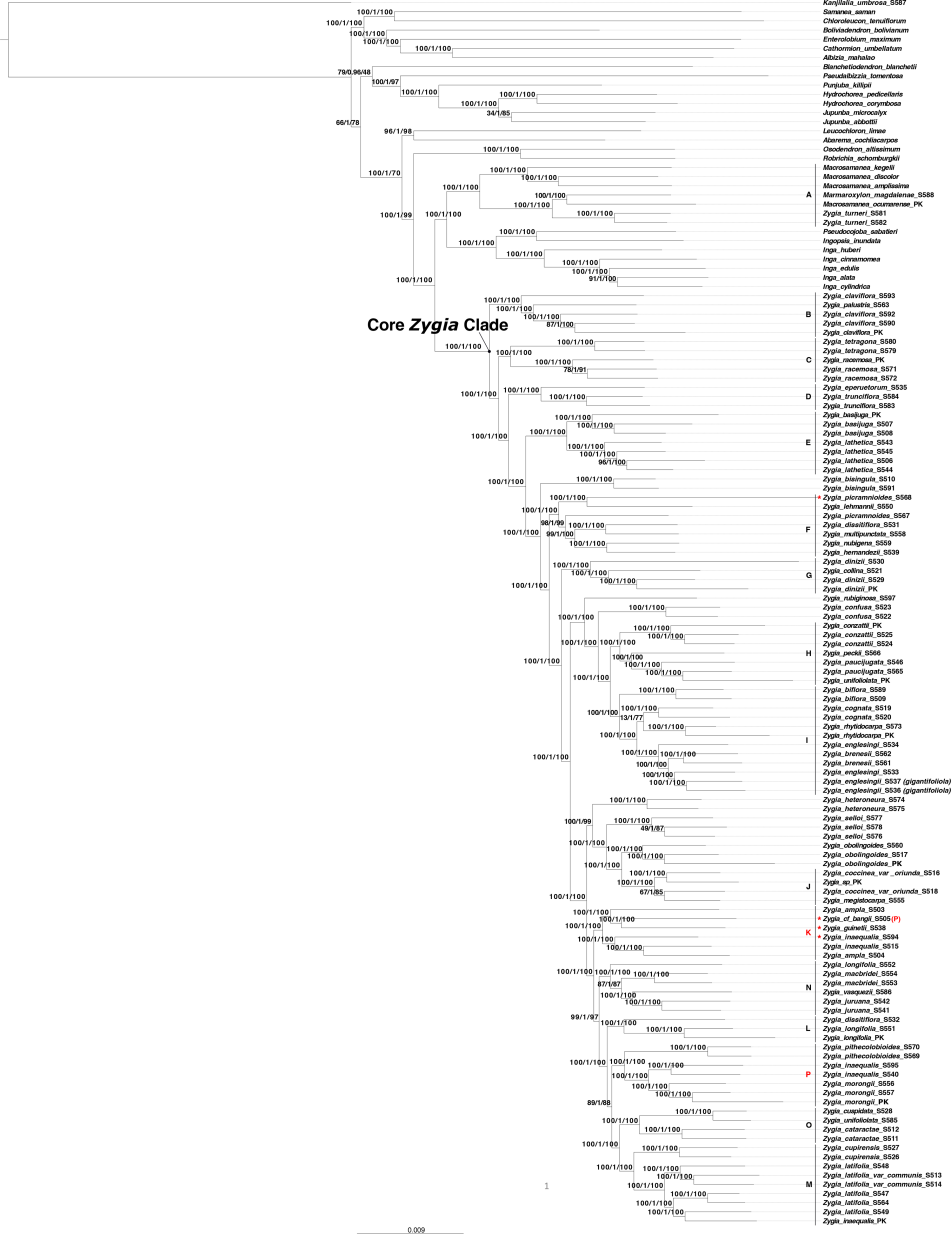
Phylogeny estimated using a maximum likelihood analysis as implemented in IQtree2 based on the concatenated dataset that includes all accessions and 1172 loci (i.e. multiple accessions for some species and a total of 134 taxa and putatively paralogous loci and loci with ≥50% missing data or ≥50% missing taxa excluded, as well as the loci not suitable for analysis in IQtree2). Support values at nodes are SH-aLRT support from the likelihood ratio test (SHS), approximate posterior probability (APP) and ultrafast bootstrap support (UBS), in that order (SHS/APP/UBS). Differences in topology compared to **Figure 2** are marked in red.

## 2 Materials and methods

### 2.1 Taxon sampling and DNA extraction

We included 134 accessions in total. Leaf samples were collected in the field and preserved in silica gel or sampled from herbarium specimens. DNA was extracted from 92 accessions of *Zygia*, *Marmaroxylon magdalenae*, and *Kanjilalia umbrosa* (Wall.) Sanjappa & Pusalkar using the QIAGEN DNeasy Plant Mini Kit following the instructions from the manufacturer (Qiagen, 2025). Additional DNA sequence data from previous studies (Nicholls et al., 2015; Koenen et al., 2020; Ringelberg et al., 2022) were obtained from EMBL-EBI European Nucleotide Archive (ENA, 2025). Raw data were downloaded for accessions representing 12 species of *Zygia* and 28 species of 20 other mimosoid genera. For a full overview of the sampling of taxa, and collection and voucher information for samples used for DNA sequencing in the present study, see **Supplementary Table 1**.

### 2.2 DNA sequencing

The DNA samples were sent to the University of Exeter DNA Sequencing Facility (Exeter, UK) for DNA library preparation, enrichment, and sequencing as described in Schley et al. (2025). A total of 1,320 low-copy nuclear loci were targeted using a bait set specifically designed for mimosoid legumes: “Mimobaits” (Nicholls et al., 2015; Koenen et al., 2020).

### 2.3 DNA sequence assembly

The quality of the raw sequencing reads was examined using FastQC 0.12.1 (Andrews, 2010). The results from FastQC for all individuals were aggregated into one single report using MultiQC 1.25 (Ewels et al., 2016), and the results were inspected.

The sequencing reads were trimmed using Trimmomatic 0.39 (Bolger et al., 2014) with the following settings: LEADING: 28, TRAILING: 28, SLIDINGWINDOW: 4:30, MINLEN: 36. Low-quality bases (quality score, *Q* ≤ 28) were removed from both the 5′ and the 3′ end, a sliding window of 4 bases was used, and the bases were trimmed when the average quality of the window dropped below 30 (*Q* < 30). Reads shorter than 36 bases after trimming were discarded. FastQC 0.12.1 (Andrews, 2010) was run again, on the trimmed reads, in order to see potential improvement from the trimming.

The trimmed reads were assembled in Hybpiper 2.3.2 (Johnson et al., 2016) using the 1,320 targeted “Mimobaits” loci as a reference. Commands run in Hybpiper were “assemble”, which matches all sequencing reads to the reference targets and assembles them into contigs on a per-gene basis, i.e., in our case, it mainly creates assembled exons but will also recover introns and spacer regions; “Stats”, which produces a summary table of the samples and the target genes; “Recovery heatmap”, which creates a heatmap showing all samples and genes obtained for each accession; “Retrieve sequences”, which creates one alignment file for each targeted locus (in this study exons) including all accessions with data recovered for that gene; and “paralog retriever”, which provides information on putatively paralogous loci. Hybpiper was first run with multiple accessions for selected species of *Zygia*. The recovery heatmap provided information on coverage of data for each accession, and based on that, one accession per species was chosen for a second run in Hybpiper with reduced taxon sampling.

### 2.4 Dataset generation

The locus files were aligned with MAFFT 7.526 (Katoh and Standley, 2013) using a high-accuracy multiple sequence alignment strategy with the following settings: --localpair --maxiterate 1000 --adjustdirectionaccurately. The Smith–Waterman algorithm was used for all pairwise comparisons among sequences, which is more accurate than the default. The alignments were iteratively refined up to 1,000 times and sequences in reverse complement orientation were automatically flipped.

Statistics were run for all aligned loci using the AMAS—Alignment Manipulation And Summary tool 1.0 (Borowiec, 2016), which provided information on, e.g., number of taxa, alignment length, and missing data percentage in each locus alignment. One statistics summary was created for the loci containing one accession of each species and another was created for the loci containing all accessions.

The locus alignment files were trimmed using trimAl 1.5.rev0 (Capella-Gutiérrez et al., 2009) and the following settings: -automated -resoverlap 0.65 -seqoverlap 0.65. Columns were kept when at least 65% of the sequences had a valid residue, i.e., not a gap at that position. Sequences were kept when at least 65% of their positions overlapped with columns that had passed the previous filtering, and sequences with less than 65% coverage in the final trimmed alignment were removed.

### 2.5 Tree inference and species tree assembly

IQtree2 2.3.6 (Minh et al., 2020) was used to infer gene trees based on each locus alignment containing one accession per species and from the data in each locus alignment including all accessions. IQtree2 was run using the settings -m MFP -B 1000 -bnni. The best-fitting nucleotide substitution model was automatically chosen using ModelFinder (Kalyaanamoorthy et al., 2017). Ultrafast bootstrapping was run with 1,000 replicates, and an extra nearest-neighbor interchange search optimized bootstrap trees, which prevents overestimating support values.

Gene trees obtained from IQtree2 were used to infer species trees with the multispecies coalescent model (MSC) as implemented in Astral-Pro3 (Zhang et al., 2020) and Astral-III (Zhang et al., 2018) using the -t2 setting [for full annotation of the phylogeny, e.g., quartet support values and local posterior probabilities (LPP)]. Gene trees produced from putatively paralogous loci, and gene trees from loci with ≥50% missing data or ≥50% missing taxa, were either included or excluded for the four species trees assembled using Astral-III. Information on putatively paralogous loci was obtained as part of the output from Hybpiper (i.e., from using the command paralog retriever) and information on missing data and missing taxa from the statistics summary from AMAS. For the species trees assembled using Astral-Pro3, all loci were included since Astral-Pro3 can handle ILS and allow multi-copy genes.

To visualize the amount of concordance between different gene trees in our species trees, quartet scores calculated in Astral-III (Zhang et al., 2018) for datasets #a and #b were plotted as pie charts on nodes using R 4.4.2 (R Core Team, 2024) and the packages ape 5.8.1 (Paradis and Schliep, 2019), treeio 1.30.0 (Wang et al., 2020), and phytools 2.4.4 (Revell, 2024). Quartet scores were also calculated for internal nodes for datasets #c and #d, but those results are provided as numbers on nodes.

A polytomy test, i.e., a statistical test that evaluates if a polytomy can be rejected and the node is a “true split”, was run using the -t 10 setting in Astral-III (Zhang et al., 2018).

### 2.6 Concatenated datasets

The aligned loci containing all accessions were also concatenated into two different super matrices using pxcat 1.1 from phyx 1.3.2 (Brown et al., 2017). One concatenated dataset included all loci obtained from Hybpiper, and the other concatenated dataset had putatively paralogous loci and loci with ≥50% missing data or ≥50% missing taxa excluded. The concatenated datasets were trimmed with trimAl 1.5.rev0 (Capella-Gutiérrez et al., 2009) using the setting automated1, which lets trimAl automatically decide the best trimming strategy for the alignment and chooses one of the in-built trimming modes. The trimmed concatenated datasets were analyzed within the maximum likelihood (ML) framework in IQtree2 2.3.6 (Minh et al., 2020). The settings used for calculating support values on the resulting phylogeny were as follows: -alrt 1000 -abayes -B 1000, which runs the Shimodaira–Hasegawa approximate likelihood ratio test with 1,000 replicates, runs the approximate Bayes test for each branch, and runs ultrafast bootstrap with 1,000 replicates. Support values obtained were SH-aLRT support from the likelihood ratio test (SHS), approximate posterior probability (APP), and ultrafast bootstrap support (UBS), presented on nodes in that order (SHS/APP/UBS).

### 2.7 Comparison of phylogenetic trees and gene tree concordance to species trees

The phylogenetic trees were inspected in Figtree 1.4.4 (Rambaut, 2018) and visually compared to investigate if different datasets and ways of handling potential paralogs as well as missing data had an impact on topology. Topological differences were also investigated using the Robinson–Foulds (RF) statistics, which was calculated using IQtree2 (Minh et al., 2020) or R 4.4.2 (R Core Team, 2024) and the packages ape 5.8.1 (Paradis and Schliep, 2019) and phangorn 2.12.1 (Schliep, 2011). Normalized quartet scores (NQS) were calculated for species trees in Astral-III (Zhang et al., 2018) and Astral-Pro3 (Zhang et al., 2020) in order to evaluate to what extent the species trees agree with the gene trees. The phylogenetic trees were designed using Inkscape 1.3.2 (“Inkscape,” 2025).

## 3 Results

### 3.1 Dataset generation

Summary statistics for aligned loci (one for the loci with one accession per species and one for the loci including all accessions) were used to identify alignments that had ≥50% missing taxa or ≥50% missing data and to compare the number of parsimony informative sites (PIS) among the different datasets. The results show that the average number of PIS is the lowest when one accession per species was sampled, indicating sequence variation within species (**Table 1**). Hybpiper provided information on loci that were putatively paralogous.

**Table 1:** Alignment statistics.

Dataset	No. of loci^1^	Total no. of sites	Total no. of PIS	Average no. of PIS/locus
#a) Reduced taxon sampling (one accession/species, loci curated^1^)	1,190	2,348,184	294,632	248
#b) All accessions, loci curated^1^	1,172	2,339,993	383,863	328
#c) Reduced taxon sampling (one accession/species, all loci)	1,314	2,577,139	320,727	244
#d) Standard (all accessions, all loci)	1,315	2,624,237	423,192	322
#e) Concatenated (all accessions, loci curated^1^)	1,172	2,052,673	363,729	310
#f) Concatenated alignment (all accessions, all loci)	1,315	2,195,184	394,798	300

^1^ Putatively paralogous loci and loci with ≥ 50% missing data or taxa excluded.

Number of parsimony informative sites (PIS) for the included datasets.

### 3.2 Phylogenetic tree inference

For the loci with all accessions, 1,311 gene trees were obtained after running IQtree2, and for the loci with one accession per species, 1,312 gene trees were obtained after running IQtree2 (**Table 2**). Two locus alignments contained fewer than three sequences, which is below the number of sequences needed for IQtree2 to operate, and two loci alignments contained too much ambiguous data. The datasets were analyzed using several different approaches, and in total, eight phylogenetic trees were generated. A summary of the phylogenetic trees and key statistics is provided in **Table 2**. Species trees and phylogenetic trees based on concatenated datasets were rooted on *K. umbrosa*. *K. umbrosa* is closely related to *Sanjappa cynometroides* E.R.Souza & Krishnaraj (Thulin, 2024) and, hence, not part of the same monophyletic group as the remaining taxa in this study (Ferm et al., 2021).

**Table 2:** Summary and key statistics on the phylogenetic trees.

Dataset	Phylogenetic tree	No. of gene trees/loci	Gene tree curation^1^	No of taxa	NQS score	Model/software
#b	**Figure 2**	1,172	Excluded	134	0.6	MSC/Astral-III
#e	**Figure 3**	1,172	Excluded	134	–	ML/IQ-tree2
#a	**Supplementary Figure 1**	1,190	Excluded	79	0.7	MSC/Astral-III
#c	**Supplementary Figure 2**	1,312	Included	79	0.66	MSC/Astral-III
#c	**Supplementary Figure 3**	1,312	Included	79	0.66	MSC/Astral-Pro3
#d	**Supplementary Figure 4**	1,311	Included	134	0.6	MSC/Astral-III
#d	**Supplementary Figure 5**	1,311	Included	134	0.6	MSC/Astral-Pro3
#f	**Supplementary Figure 6**	1,315^2^	Included	134	–	ML/IQ-tree2

^1^ Gene trees from putatively paralogous loci and/or loci with ≥50% missing data or taxa: included/excluded.

^2^ Loci that did not generate gene trees in IQtree2 were also included in the concatenated dataset #f.

All phylogenetic trees show basically the same topology when compared visually (**Figures 2**, **3**; **Supplementary Figures 1**−**6**). Thus, the same topology, with few small differences, is obtained when analyzing datasets with or without putatively paralogous loci, including or excluding loci containing ambiguous characters or large amounts of missing data or taxa. These indications are confirmed by normalized RF statistics, which show that tree topologies with few differences are obtained (Robinson and Foulds, 1981; **Supplementary Table 2**). Moreover, the species tree based on the MSC (**Figure 2**) and the phylogenetic tree based on ML analyses of the concatenated dataset (**Figure 3**) differ in three accessions having different positions only, which is also reflected in the normalized RF statistics (**Supplementary Table 2**).

Within *Zygia*, 27 out of 100 branches are present in the majority of gene trees (**Figure 2**), and the NQS scores (**Table 2**) show that approximately 60% to 70% of the quartets in the species trees are consistent with the quartets in the gene trees. However, high levels of gene tree discordance are present within *Zygia* (**Figure 2**), with most quartet frequencies being equal for the three different topologies or nearly so. A few nodes show strongly unequal quartet frequencies for the two alternative topologies.

The polytomy tests show that approximately 25% of the nodes within the core *Zygia* clade were better regarded as polytomies.

### 3.3 Taxonomic limits of *Zygia*

*Zygia* is non-monophyletic since one species, *Zygia turneri* (McVaugh) Barneby & J.W.Grimes, groups with the clade formed by *Marmaroxylon magdalenae* and *Macrosamanea ocumarensis* (Pittier) Ferm. Together, these three species are the sister group to the remaining species of *Macrosamanea* (**Figures 2**, **3**: clade A). The core *Zygia* clade is strongly supported and shown to be sister to a *Macrosamanea*–*Inga*–*Ingopsis*–*Pseudocojoba* clade (including *Z. turneri* and *Marmaroxylon magdalenae*). These results are strongly supported (**Figure 2**: LPP = 1; **Figure 3**: SHS = 100, APP = 1, and UBS = 100) and present in a clear majority of gene trees (**Figure 2**).

### 3.4 Species relationships within *Zygia*

Within the core *Zygia* clade, relationships between species are for the most part strongly supported by SHS, APP, and UBS values (**Figure 3**), but LPP values vary and gene tree discordance is common (**Figure 2**). Most species are resolved as monophyletic, but some are paraphyletic with other species nested within them, e.g., *Z*. *claviflora* and *Z*. *palustris* (**Figures 2**, **3**: Clade B), or polyphyletic with accessions representing the same species found in different parts of the tree, e.g., *Z*. *dissitiflora* (**Figures 2**, **3**: Clades F and L).

## 4 Discussion

This study investigates the evolutionary history of *Zygia* using a more comprehensive DNA sequence dataset (1,315 low-copy nuclear genes) and a larger sample of species (92 accessions representing 48 of 60 species) than any previous study (e.g., Ferm et al., 2019). The well-supported phylogenetic trees (**Figures 2**, **3**) give a clear picture of relationships allowing for a classification of *Zygia* and related genera based on monophyly, including the assignment of the species *Z*. *turneri* and *Marmaroxylon magdalenae* to *Macrosamanea*, and of *Marmaroxylon eperuetorum* to *Zygia*.

Regardless of inclusion or exclusion of putatively paralogous genes, missing data, and which inference tool was used, the same topology was recovered. This indicates that potentially paralogous genes did not mislead the results in any impactful way, a conclusion that is further supported by the normalized RF statistics (**Supplementary Table 2**) and the similar NQS values (**Table 2**) for all species trees. This is in line with previous work (Yan et al., 2022) that has shown that coalescent-based methods such as Astral are robust to species tree inference based on datasets that include paralogs.

Some branches within *Zygia* are supported by most gene trees, but many branches show high levels of gene tree discordance (**Figure 2**). Gene tree discordance is commonly detected in multigene studies and can occur for different biological reasons, for example, ILS, hybridization/introgression, and gene duplication/loss (Maddison, 1997), but it may also occur because of gene tree error. We have mitigated possible gene tree error by including large numbers of loci, by excluding potentially paralogous genes, and by data trimming. In addition, we conducted concatenated analyses that better deal with potentially limited phylogenetic information in individual genes. The fact that a polytomy cannot be rejected for approximately 25% of the nodes within the core *Zygia* clade could be due to gene tree error but is also consistent with the occurrence of ILS. In the core *Zygia* clade, quartet frequencies are often nearly equal for the two alternative topologies, which indicate the presence of ILS (Pease et al., 2018).

High levels of ILS are considered an indication of a relatively short time between speciation events (Maddison, 1997; Whitfield and Lockhart, 2007), and the frequent occurrence of ILS throughout the phylogeny of *Zygia* could be due to a recent rapid radiation giving rise to many species. This is supported by the short branch lengths seen in **Figure 3**, which indicate few evolutionary changes between nodes (Maddison, 1997; Whitfield and Lockhart, 2007). Recent rapid radiations have been demonstrated in many species-rich plant groups in the Neotropics (Koenen et al., 2015) including *Inga*, one of the sister genera to *Zygia* (Richardson et al., 2001). *Inga* is similar to *Zygia* in being widespread in the Neotropics, with several species occurring throughout Amazonia (Barneby and Grimes, 1997; Pennington, 1997). For a few nodes, including at least two in the core *Zygia* clade (**Figure 2**), the quartet frequencies of the two alternative topologies are strongly unequal, which may indicate that other causes of gene tree discordance other than ILS are involved (Degnan and Rosenberg, 2009; Pease et al., 2018). For such nodes, further studies, e.g., aimed at investigating the possible presence of hybridization (and introgression), would be worthwhile (Schley et al., 2025).

### 4.1 Taxonomic limits of *Zygia*

*Zygia* is shown to be non-monophyletic since *Z*. *turneri* is nested within *Macrosamanea* (**Figures 2**, **3**: clade A). *Z. turneri* has flowers “arising from the axil of lately fallen lvs” according to Barneby and Grimes (1997); thus, it is not cauliflorous like most species of *Zygia* but more similar to species of *Macrosamanea*, which have axillary inflorescences. *Z. turneri* has leaves with one pair of pinnae and one pair of leaflets on each pinna (**Supplementary Table 3**), whereas species of *Macrosamanea* have leaves with two pairs of pinnae. However, variation in foliage formula also occurs within, e.g., *Zygia*, and the number of pinnae pairs per leaf thus might not be a reliable diagnostic character. *Z. turneri* does not have nectaries on the floral bracts as do some species of *Macrosamanea* (Barneby and Grimes, 1996); however, nectaries on the floral bracts are missing in several species of *Macrosamanea*, e.g., *M. kegelii* (*Meisn.*)Kleinh., *M. macrocalyx* (Ducke) Barneby & J.W.Grimes, and *M. ocumarensis*, and the trait could be a derived feature in some species, possibly supporting a subclade within *Macrosamanea* (Ferm and Rydin, 2024).

Given the evidence above, to render *Zygia* monophyletic, we transfer *Z*. *turneri* to *Macrosamanea*. This change extends the distribution of *Macrosamanea*, currently restricted to northern South America, further north to Mexico. There, *Z*. *turneri* (now *Macrosamanea turneri*) occurs in deciduous or palm forests at elevations up to 200 m in Jalisco and Oaxaca (**Supplementary Table 3**; Tropicos.org, 2026).

*Marmaroxylon* has been synonymized with *Zygia* (Barneby and Grimes, 1997), and species of *Marmaroxylon* have been shown to mix phylogenetically with species of *Zygia* (Ferm et al., 2019). *Marmaroxylon magdalenae* was placed as a synonym of *Z*. *ocumarensis* (Pittier) Barneby & J.W.Grimes based on morphological similarities (Barneby and Grimes, 1997), but *Z*. *ocumarensis* has since then been transferred to *Macrosamanea* and is currently recognized as *Macrosamanea ocumarensis* (Ferm and Rydin, 2024). No taxonomic change has been made for *Marmaroxylon magdalenae* since it was considered premature (Ferm et al., 2019; Ferm and Rydin, 2024). *Marmaroxylon magdalenae* is here resolved as the sister to *Macrosamanea ocumarensis* (**Figure 2**; **Figure 3**: clade A), but whereas the SHS/APP/UBS support for the sister relationship is very strong (100/1/100: **Figure 3**), the LPP is somewhat lower (0.91, 0.87: **Figure 2**). It has been suggested, however, that LPP values as low as 0.7 yield high precision and should probably not be *a priori* dismissed (Sayyari and Mirarab, 2016). *Marmaroxylon magdalenae* and *Macrosamanea ocumarensis* are both represented by only one accession each in this study, and while additional sampling would be beneficial, it will be difficult to obtain since both species are known only from a few herbarium specimens. *Marmaroxylon magdalenae* is known only from two collections, and the type collection is included here (**Supplementary Table 3**). Therefore, we choose to make the taxonomic and nomenclatural readjustment based on the current evidence and thus include *Marmaroxylon magdalenae* in *Macrosamanea* as a distinct separate species instead of as a synonym to *Macrosamanea ocumarensis*. With the exception of *Marmaroxylon eperuetorum*, all other species formerly in *Marmaroxylon* now have nomenclatural combinations in *Zygia* (Barneby and Grimes, 1997; Ferm et al., 2019), or have been shown to belong in other genera (Ferm and Rydin, 2024).

*Marmaroxylon eperuetorum* is analyzed molecularly here for the first time and is shown to be positioned within the core *Zygia* clade (**Figures 2**, **3**: clade D). Previous studies based on morphology regarded *Marmaroxylon eperuetorum* either to be closely related to *Marmaroxylon ocumarense* (now *Macrosamanea ocumarensis*) and *Marmaroxylon magdalenae* (Rico-Arce, 1991) or as distinct from all other *Zygia* species (Barneby and Grimes, 1997). Our results show, however, that while *Macrosamanea ocumarensis* and *Marmaroxylon magdalenae* are phylogenetically placed outside of *Zygia* (**Figures 2**, **3**: clade A), *Marmaroxylon eperuetorum* is closely related to *Z. trunciflora* (Ducke) L.Rico (**Figures 2**, **3**: clade D). Some reproductive features support the relationship of *Marmaroxylon eperuetorum* and *Z. trunciflora*; both have green or greenish calyces, cream or glaucous green corollas, and red or crimson stamen filaments, at least on their upper half (**Supplementary Table 3**). By contrast, *Z. trunciflora* has flowers with an intrastaminal disc (as in most species of *Zygia*) that is not found in *Marmaroxylon eperuetorum* (Barneby and Grimes, 1997). Moreover, *Marmaroxylon eperuetorum* has leaves with two pairs of pinnae, while *Z*. *trunciflora* has leaves with one pair of pinnae only (**Supplementary Table 3**; Barneby and Grimes, 1997). However, species with this variation in foliage formula are intermixed throughout the phylogeny of *Zygia* (**Figures 2**, **3**), and there is no clear evolutionary pattern to the occurrence of number of pinnae per leaf. The same variation in foliage formula occurs in *Macrosamanea*, which, based on the phylogenetic delimitations made here, includes species with two or more pairs of pinnae per leaf, or a single pinnae pair (i.e., in *Z*. *turneri*). It is evident that leaf formula is a variable character that cannot be used for identification of *Zygia* and allies. A related mimosoid genus exception is *Inga*, comprising approximately 300 species, all of which have once-pinnate leaves (Pennington, 1997). Nevertheless, clearly *Marmaroxylon eperuetorum* should be regarded as a member of *Zygia*, as *Z*. *eperuetorum* (Sandwith) Barneby & J.W.Grimes. It is worth noting that both *Marmaroxylon eperuetorum* and *Z*. *trunciflora* are poorly represented in herbaria. *Marmaroxylon eperuetorum* is endemic and locally abundant in wallaba forest in Guyana, while *Z. trunciflora* is only known from primary forest in Amazonian Brazil (**Supplementary Table 3**), indicating that targeted fieldwork is necessary to better understand these species.

### 4.2 Species relationships within *Zygia*

The sections in *Zygia*, defined based on morphology (Barneby and Grimes, 1997), are not recovered in this study. As also shown by Ferm et al. (2019), species from all sections are intermixed in the phylogeny (**Supplementary Table 4**; **Figures 2**, **3**). Moreover, five sections comprise only one species each, of which three species have been transferred to other genera (Ferm and Rydin, 2024). Of the remaining two single species sections, *Z*. *racemosa* was placed as the only species in *Zygia* sect. *Marmaroxylon* but is here shown to be the sister to *Z*. *tetragona*, a member of *Zygia* sect. *Parazygia* (**Figures 2**, **3**: Clade C). The final species with its own section is *Z*. *eperuetorum*, which is here shown to be the sister to *Z*. *trunciflora*, a member of *Zygia* sect. *Zygia* (**Figures 2**, **3**: Clade D). Clearly, *Zygia* species are difficult to classify based on morphology, and diagnostic morphological characters supporting an intrageneric classification are lacking.

Species relationships within *Zygia* are overall well-resolved and strongly supported (**Figures 2**, **3**). Thus, adding more DNA data and more accessions compared to the previous study (Ferm et al., 2019) clearly improved resolution within *Zygia*. *Zygia latifolia*, the type species of *Zygia* that was non-monophyletic in Ferm et al. (2019), is here resolved as monophyletic (**Figures 2**, **3**: Clade M), as well as *Z*. *brenesii*, *Z*. *lathetica*, and *Z*. *basijuga* (**Figures 2**, **3**: Clades E and I). However, some of the other non-monophyletic species in Ferm et al. (2019) are again recovered as non-monophyletic (*Z*. *claviflora*, *Z*. *coccinea*, *Z*. *inaequalis*, and *Z*. *unifoliolata* in **Figures 2**, **3**: Clades B, J, H, K, M, O, and P). In the core *Zygia* clade, 13 species are non-monophyletic (**Figures 2**, **3**), and in general, the reasons for this are not yet fully known. Misidentification is one possibility since most species of *Zygia* are difficult to determine, and many are even impossible to identify when sterile or when fruits are missing. Other reasons could be poorly delimited species in need of taxonomic evaluation or hybridization (and introgression). Non-monophyly in *Zygia* could also reflect an ancestor–descendant species relationship and ILS where ancestral allelic polymorphism is retained through speciation events (Naciri and Linder, 2015; Pennington and Lavin, 2016). As shown in this study, ILS seems to be common in *Zygia* (**Figure 2**).

Among non-monophyletic species in *Zygia* is *Z*. *claviflora*, which is paraphyletic with regard to *Z*. *palustris* (**Figures 2**, **3**: clade B). According to Barneby and Grimes (1997), the morphology of *Z. palustris* resembles that of *Z. claviflora*, but their fruits differentiate them. Inspection of the *Z. palustris* specimen included in the present study (**Supplementary Table 3**) shows it to have three pairs of pinnae per leaf, with many small leaflets on each pinna. The species occurs in the black-water swamp forest in Amazonian Venezuela. Other specimens of *Z. palustris*, including the holotype (**Supplementary Table 3**), adds the characters of acuminate, ca 2-cm-long leaflets, white−pink flowers, and bi-colored stamen filaments that are white basally and pink distally. By contrast, the leaflets of *Z. claviflora* are more rounded apically and the flowers are white−green−yellow according to collection field labels, including on a syntype inspected online (**Supplementary Table 3**). According to Tropicos.org (Tropicos.org, 2026), both species have been reported from the same general localities in Venezuela and Guyana, but the ecology of *Z. palustris* is always described as swampy, which is never the case for *Z. claviflora*. Thus, *Z. palustris* and *Z. claviflora* differ in leaflet shape, flower color, and ecology. Additional samples of *Z. palustris* should be included in future studies to confirm, or disprove, its status as a separate species. Another apparent example of paraphyly is *Z*. *dinizii*, which is paraphyletic with regard to *Z*. *collina* (**Figures 2**, **3**: clade G), but these two species are morphologically identical based on current knowledge (the fruits of *Z*. *dinizii* are unknown). Misidentification of the specimen representing *Z*. *collina* cannot be ruled out or, perhaps, *Z*. *collina* should be synonymized under *Z*. *dinizii*, but additional specimens of *Z*. *collina* are needed to further investigate the distinctiveness of these two species.

## 5 Conclusions

Based on coalescent-based and concatenated analyses of robust datasets comprising more than 100 accessions and over 1,300 putatively independently evolving low-copy nuclear genes, many evolutionary relationships in *Zygia* and relationships to closely related mimosoid genera are resolved. Taxonomic realignments proposed here render *Zygia* as monophyletic: *Z. turneri* and *M. magdalenae* are transferred to *Macrosamanea*, with which they better agree morphologically. These taxonomic changes result in a strongly supported *Zygia* clade.

High levels of gene tree discordance are demonstrated for most nodes in the core *Zygia* clade, most likely due to ILS being common in the genus. These conclusions are consistent with a recent and rapid radiation in *Zygia*. Some species are shown to be non-monophyletic, and further studies that include additional samples of those species and evaluation of morphology are needed to assess their taxonomic status.

## 6 Taxonomic treatments

The species *Z. turneri* and *M. magdalenae* are more closely related to the genus *Macrosamanea* than to *Zygia*. We propose their inclusion in *Macrosamanea*, which will render both *Zygia* and *Macrosamanea* monophyletic.

*Macrosamanea turneri* (McVaugh) Ferm comb.nov.

Basionym: *Pithecellobium turneri* McVaugh, Fl. Novo-Galiciana 5: 238 (1987). MEXICO. Hills between Bahía Navidad and La Manzanilla on Bahía Tenacatita; tropical deciduous forest with *Ficus*, *Aralia*; *Trichilia*, *Bursea*; elevation 150−200 m; 12 Nov 1960; *McVaugh* 20980 (Holotype MICH; Isotypes CAS, MO, NY). *Z. turneri* (McVaugh) Barneby & J.W.Grimes, *Mem. New York Bot. Gard.* 74(2): 117 (1997).

*Macrosamanea magdalenae* (Killip ex L.Rico) Ferm comb.nov.

Basionym: *M. magdalenae* Killip ex L.Rico, *Kew Bull.* 46(3): 516 (1991). COLOMBIA. Santander, Vicinity of Barranca Bermeja, Magdalena Valley between Sogamoso and Carare Rivers; 100−500 m; without date; *Haught* 2097 (Holotype K; Isotypes S, US).

## Data Availability

The datasets presented in this study can be found in online repositories. The names of the repository/repositories and accession number(s) can be found below: https://www.ebi.ac.uk/ena, PRJEB108520, http://www.datadryad.org, 1c59zw4bm.

## References

[B14] (2025). Inkscape. Available online at: https://www.inkscape.org (Accessed April 29, 2026).

[B1] Andrews S. (2010). FastQC: a quality control tool for high throughput sequence data. Available online at: https://cir.nii.ac.jp/crid/1370584340724053142 (Accessed June 30, 2025).

[B2] Barneby R. Grimes J. (1996). “ Silk tree, guanacaste, monkey’s earring. Part I. Abarema, albizia, and allies,” in Memoirs of the new york botanical garden. (New York: New York Botanical Garden), vol. 74, 1–292.

[B3] Barneby R. C. Grimes J. W. (1997). “ Silk tree, guanacaste, monkey’s earring: a generic system for the synandrous Mimosaceae of the Americas. Part II. Pithecellobium, Cojoba and Zygia,” in Memoirs of the new york botanical garden. (New York: New York Botanical Garden).

[B4] Bolger A. M. Lohse M. Usadel B. (2014). Trimmomatic: a flexible trimmer for Illumina sequence data. Bioinformatics 30, 2114–2120. doi: 10.1093/bioinformatics/btu170 24695404 PMC4103590

[B5] Borowiec M. L. (2016). AMAS: a fast tool for alignment manipulation and computing of summary statistics. PeerJ 4, e1660. doi: 10.7717/peerj.1660 26835189 PMC4734057

[B6] Brown J. W. Walker J. F. Smith S. A. (2017). Phyx: phylogenetic tools for unix. Bioinformatics 33, 1886–1888. doi: 10.1093/bioinformatics/btx063 28174903 PMC5870855

[B7] Capella-Gutiérrez S. Silla-Martínez J. M. Gabaldón T. (2009). trimAl: a tool for automated alignment trimming in large-scale phylogenetic analyses. Bioinformatics 25, 1972–1973. doi: 10.1093/bioinformatics/btp348 19505945 PMC2712344

[B8] Degnan J. H. Rosenberg N. A. (2009). Gene tree discordance, phylogenetic inference and the multispecies coalescent. Trends Ecol. Evol. 24, 332–340. doi: 10.1016/j.tree.2009.01.009 19307040

[B9] ENA (2025). ebi.ab.uk/ena.

[B10] Ewels P. Magnusson M. Lundin S. Käller M. (2016). MultiQC: summarize analysis results for multiple tools and samples in a single report. Bioinformatics 32, 3047–3048. doi: 10.1093/bioinformatics/btw354 27312411 PMC5039924

[B11] Ferm J. Korall P. Lewis G. P. Ståhl B. (2019). Phylogeny of the Neotropical legume genera Zygia and Marmaroxylon and close relatives. Taxon. 68, 661–672. doi: 10.1002/tax.12117 41531421

[B12] Ferm J. Ståhl B. Wikström N. Rydin C. (2021). Phylogeny of the ingoid clade (Caesalpinioideae, Fabaceae), based on nuclear and plastid data. bioRxiv, 2021.11.23.469677. doi: 10.1101/2021.11.23.469677 38621210

[B13] Ferm J. Rydin C. (2024). Taxonomic adjustments in Mimoseae (Fabaceae): new genera described to accommodate Zygia/Inga inundata and Zygia sabatieri, and a recombination of Marmaroxylon ocumarense. Bot. J. Linn. Soc 208, boae078. doi: 10.1093/botlinnean/boae078 40388063

[B15] Johnson M. G. Gardner E. M. Liu Y. Medina R. Goffinet B. Shaw J. A. . (2016). HybPiper: Extracting coding sequence and introns for phylogenetics from high-throughput sequencing reads using target enrichment. Appl. Plant Sci. 4, 1600016. doi: 10.3732/apps.1600016 27437175 PMC4948903

[B16] Kalyaanamoorthy S. Quang Minh B. Wong T. K. F. von Haeseler A. Jermiin L. S. (2017). ModelFinder: fast model selection for accurate phylogenetic estimates. Nat. Methods 14, 587–589. doi: 10.1038/nmeth.4285 28481363 PMC5453245

[B17] Katoh K. Standley D. M. (2013). MAFFT multiple sequence alignment software version 7: improvements in performance and usability. Mol. Biol. Evol. 30, 772–780. doi: 10.1093/molbev/mst010 23329690 PMC3603318

[B18] Koenen E. J. M. Clarkson J. J. Pennington J. D. Chatrou L. W. (2015). Recently evolved diversity and convergent radiations of rainforest mahoganies (Meliaceae) shed new light on the origins of rainforest hyperdiversity. New Phytol. 207, 327–339. doi: 10.1111/nph.13490 26053172

[B19] Koenen E. J. M. Kidner C. de Souza É. R. Simon M. F. Iganci J. R. Nicholls J. A. . (2020). Hybrid capture of 964 nuclear genes resolves evolutionary relationships in the mimosoid legumes and reveals the polytomous origins of a large pantropical radiation. Am. J. Bot. 107, 1710–1735. doi: 10.1002/ajb2.1568 33253423 PMC7839790

[B21] Maddison W. P. (1997). Gene trees in species trees. Syst. Biol. 46, 523–536. doi: 10.1093/sysbio/46.3.523

[B22] Minh B. Q. Schmidt H. A. Chernomor O. Schrempf D. Woodhams M. D. von Haeseler A. . (2020). IQ-TREE 2: new models and efficient methods for phylogenetic inference in the genomic era. Mol. Biol. Evol. 37, 1530–1534. doi: 10.1093/molbev/msaa015 32011700 PMC7182206

[B23] Naciri Y. Linder H. P. (2015). Species delimitation and relationships: The dance of the seven veils. Taxon. 64, 3–16. doi: 10.12705/641.24 36611075

[B24] Nicholls J. A. Pennington T. R. Koenen E. J.M. Hughes C. E. Hearn J. Bunnefeld L. . (2015). Using targeted enrichment of nuclear genes to increase phylogenetic resolution in the neotropical rain forest genus Inga (Leguminosae: Mimosoideae). Front. Plant Sci. 6. doi: 10.3389/fpls.2015.00710 26442024 PMC4584976

[B25] Paradis E. Schliep K. (2019). ape 5.0: an environment for modern phylogenetics and evolutionary analyses in R. Bioinformatics 35, 526–528. doi: 10.1093/bioinformatics/bty633 30016406

[B26] Pease J. B. Brown J. W. Walker J. F. Hinchliff C. D. Smith S. A. (2018). Quartet Sampling distinguishes lack of support from conflicting support in the green plant tree of life. Am. J. Bot. 105, 385–403. doi: 10.1002/ajb2.1016 29746719

[B28] Pennington T. D. (1997). The genus Inga: botany. (Kew, Richmond, Royal Botanic Gardens).

[B27] Pennington R. T. Lavin M. (2016). The contrasting nature of woody plant species in different neotropical forest biomes reflects differences in ecological stability. New Phytol. 210, 25–37. doi: 10.1111/nph.13724 26558891

[B29] Qiagen (2025). Available online at: https://www.qiagen.com/us/products/discovery-and-translational-research/dna-rna-purification/dna-purification/genomic-dna/dneasy-plant-kits?catno=69106 (Accessed January 04, 2025).

[B31] Rambaut A. (2018). F igTree v.1.4.4. Available online at: https://github.com/rambaut/figtree (Accessed April 29, 2026).

[B30] R Core Team (2024). R: A language and environment for statistical computing. R Foundation for Statistical Computing. (Vienna, Austria). Available online at: https://www.R-project.org/ (Accessed June 30, 2025).

[B32] Revell L. J. (2024). phytools 2.0: an updated R ecosystem for phylogenetic comparative methods (and other things). PeerJ 12, e16505. doi: 10.7717/peerj.16505 38192598 PMC10773453

[B33] Richardson J. E. Pennington R. T. Pennington T. D. Hollingsworth P. M. (2001). Rapid diversification of a species-rich genus of neotropical rain forest trees. Science 293, 2242–2245. doi: 10.1126/science.1061421 11567135

[B34] Rico M. L. (1994). Four new species of zygia (Leguminosae: mimosoideae). Kew Bull. 49, 547–554. doi: 10.2307/4114482

[B35] Rico-Arce M. L. (1991). New species, combinations and synonyms for zygia, cojoba, marmaroxylon and pithecellobium (Leguminosae: mimosoideae, ingeae). Kew Bull. 46, 493–521. doi: 10.2307/4110539

[B36] Ringelberg J. J. Koenen E. J. M. Iganci J. R. de Queiroz L. P. Murphy D. J. Gaudeul M. . (2022). Phylogenomic analysis of 997 nuclear genes reveals the need for extensive generic re-delimitation in Caesalpinioideae (Leguminosae). PhytoKeys 205, 3. doi: 10.3897/phytokeys.205.85866 36762007 PMC9848904

[B37] Robinson D. F. Foulds L. R. (1981). Comparison of phylogenetic trees. Math. Biosci. 53, 131–147. doi: 10.1016/0025-5564(81)90043-2

[B38] Sayyari E. Mirarab S. (2016). Fast coalescent-based computation of local branch support from quartet frequencies. Mol. Biol. Evol. 33, 1654–1668. doi: 10.1093/molbev/msw079 27189547 PMC4915361

[B39] Schley R. J. Piñeiro R. Nicholls J. A. Pezzini F. F. Farbos A. Lewis G. P. . (2025). Rampant reticulation in a rapid radiation of tropical trees—Insights from inga (Fabaceae). Syst. Biol. 74, syaf027. doi: 10.1093/sysbio/syaf027 40319371 PMC12712335

[B40] Schliep K. P. (2011). phangorn: phylogenetic analysis in R. Bioinformatics 27, 592–593. doi: 10.1093/bioinformatics/btq706 21169378 PMC3035803

[B41] Ståhl B. De L. Rico-Arce M. Lewis G. P. (2010). Zygia nubigena sp. nov. (Leguminosae–Mimosoideae) from a submontane cloud forest in western Ecuador. Nord. J. Bot. 28, 453–456. doi: 10.1111/j.1756-1051.2010.00829.x 40046247

[B44] Tropicos.org . (2026) Missouri Botanical Garden. Available online at: https://tropicos.org (Accessed April 29, 2026).

[B42] Thulin M. (2024). Studies of Asian ‘Calliandra’ lead to expansion of Sanjappa (Fabaceae-Caesalpinioideae). Nord. J. Bot. 2024, e04241. doi: 10.1111/njb.04241 40046247

[B45] Vargas W. G. Moreno J. S. (2024). Zygia nataliagomezae (Fabaceae: Leguminosae). a new species from the transitional dry forest in the central Andes in the department of Valle del Cauca, Colombia. Harvard Pap. Bot. 29, 341–348. doi: 10.3100/hpib.v29iss2.2024.n14 33567615

[B46] Wang L.-G. Lam T. T.-Y. Xu S. Dai Z. Zhou L. Feng T. . (2020). Treeio: an R package for phylogenetic tree input and output with richly annotated and associated data. Mol. Biol. Evol. 37, 599–603. doi: 10.1093/molbev/msz240 31633786 PMC6993851

[B47] Whitfield J. B. Lockhart P. J. (2007). Deciphering ancient rapid radiations. Trends Ecol. Evol. 22, 258–265. doi: 10.1016/j.tree.2007.01.012 17300853

[B48] Yan Z. Smith M. L. Du P. Hahn M. W. Nakhleh L. (2022). Species tree inference methods intended to deal with incomplete lineage sorting are robust to the presence of paralogs. Syst. Biol. 71, 367–381. doi: 10.1093/sysbio/syab056 34245291 PMC8978208

[B49] Zhang C. Rabiee M. Sayyari E. Mirarab S. . (2018). ASTRAL-III: polynomial time species tree reconstruction from partially resolved gene trees. BMC Bioinf. 19, 153. doi: 10.1186/s12859-018-2129-y 29745866 PMC5998893

[B50] Zhang C. Scornavacca C. Molloy E. K. Mirarab S. (2020). ASTRAL-pro: quartet-based species-tree inference despite paralogy. Mol. Biol. Evol. 37, 3292–3307. doi: 10.1093/molbev/msaa139 32886770 PMC7751180

