# Supplementary material for: Improved phylogenetic resolution within the Neotropical rainforest genus Zygia (Mimoseae, Fabaceae) using phylogenomic data
Source: Front Plant Sci. 2026 Jun 12;17:1816329. doi: 10.3389/fpls.2026.1816329 (PMC13303398; doi:10.3389/fpls.2026.1816329)
Supplement: Supplementary file 1 [file SupplementaryFile1.zip › Supplementary_Fig.1_with_caption_Ferm_et_al_2026.pdf]

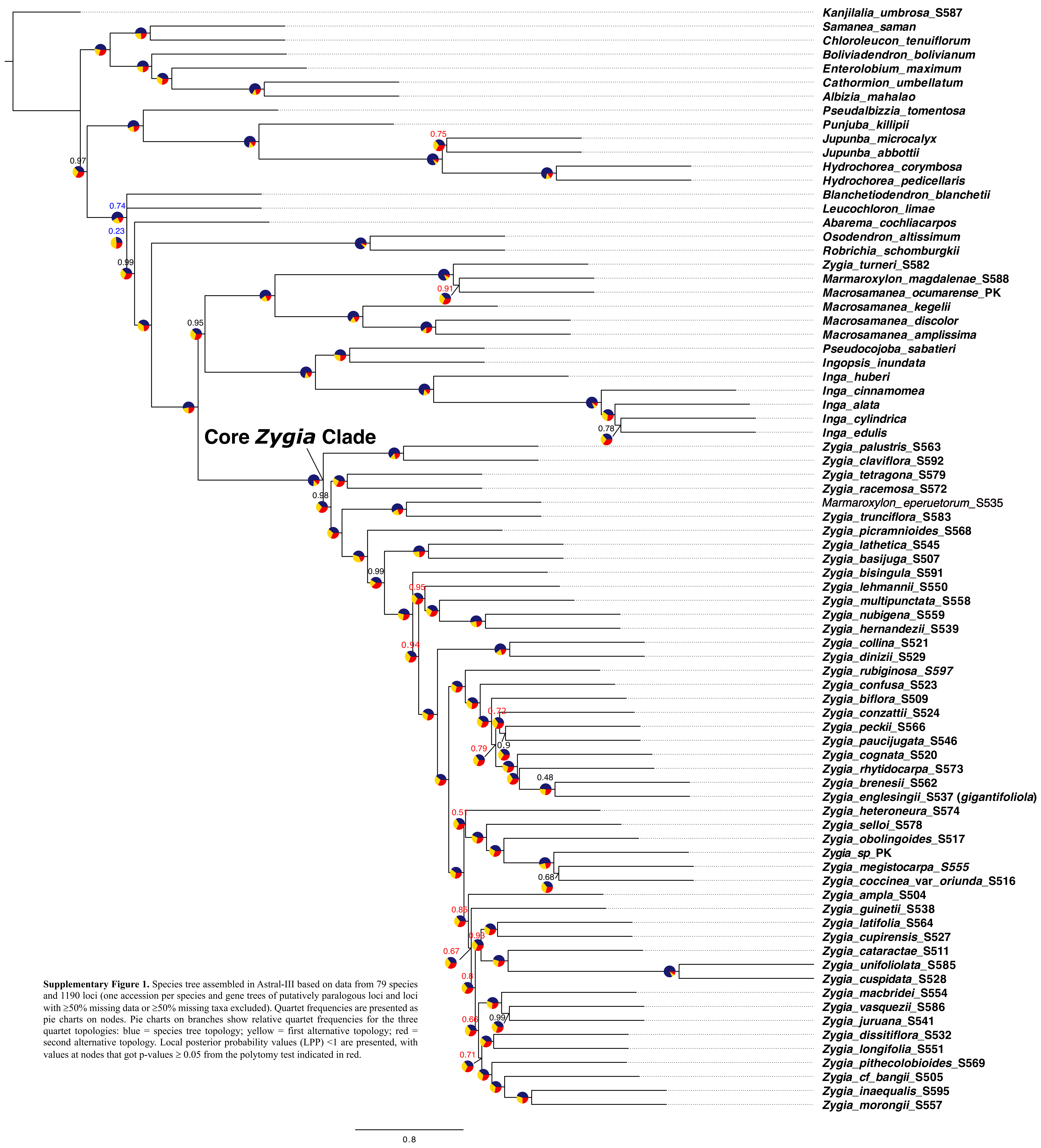

Supplementary Figure 1. Species tree assembled in Astral-III based on data from 79 species and 1190 loci (one accession per species and gene trees of putatively paralogous loci and loci with ≥50% missing data or ≥50% missing taxa excluded). Quartet frequencies are presented as pie charts on nodes. Pie charts on branches show relative quartet frequencies for the three quartet topologies: blue = species tree topology; yellow = first alternative topology; red = second alternative topology. Local posterior probability values (LPP) <1 are presented, with values at nodes that got p-values ≥ 0.05 from the polytomy test indicated in red.
