# Supplementary figures and images for: Improved phylogenetic resolution within the Neotropical rainforest genus Zygia (Mimoseae, Fabaceae) using phylogenomic data
Source: Front Plant Sci. 2026 Jun 12;17:1816329. doi: 10.3389/fpls.2026.1816329 (PMC13303398; doi:10.3389/fpls.2026.1816329)

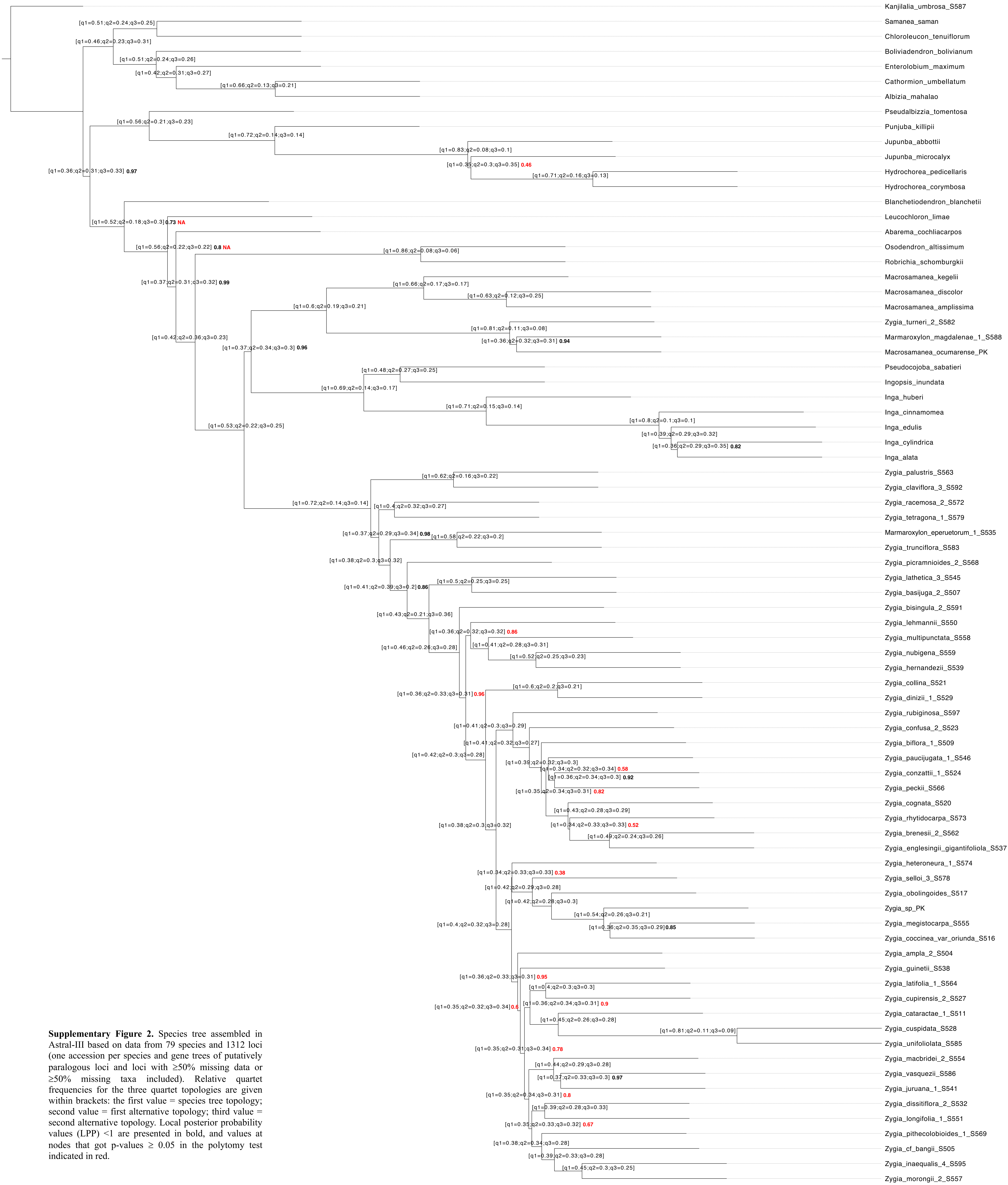

Supplement: Supplementary file 1 [file SupplementaryFile1.zip › Supplementary_Fig.2_with_captions_Ferm_et_al_2026.pdf]

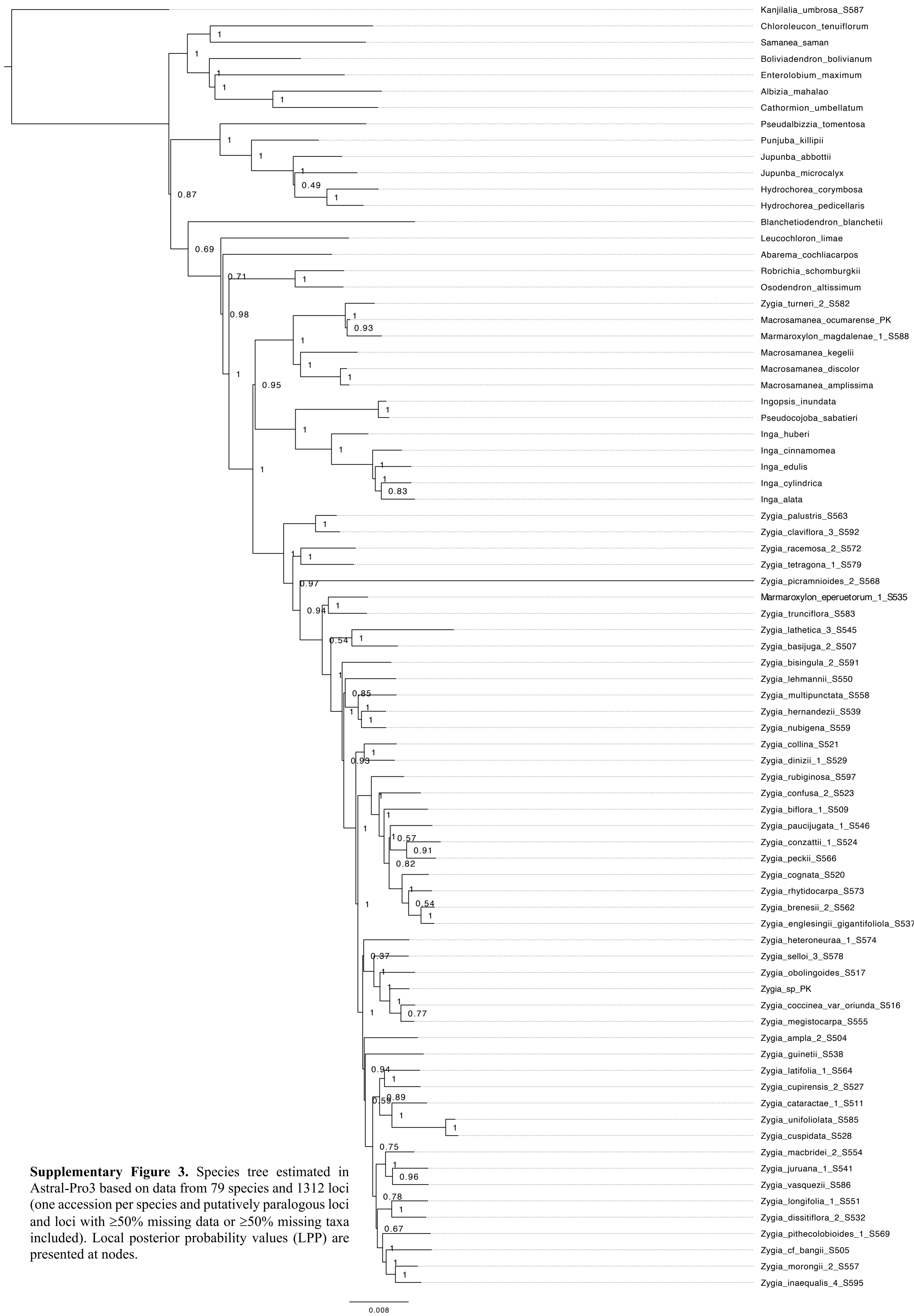

Supplement: Supplementary file 1 [file SupplementaryFile1.zip › Supplementary_Fig.3_with_caption_Ferm_et_al_2026.pdf]

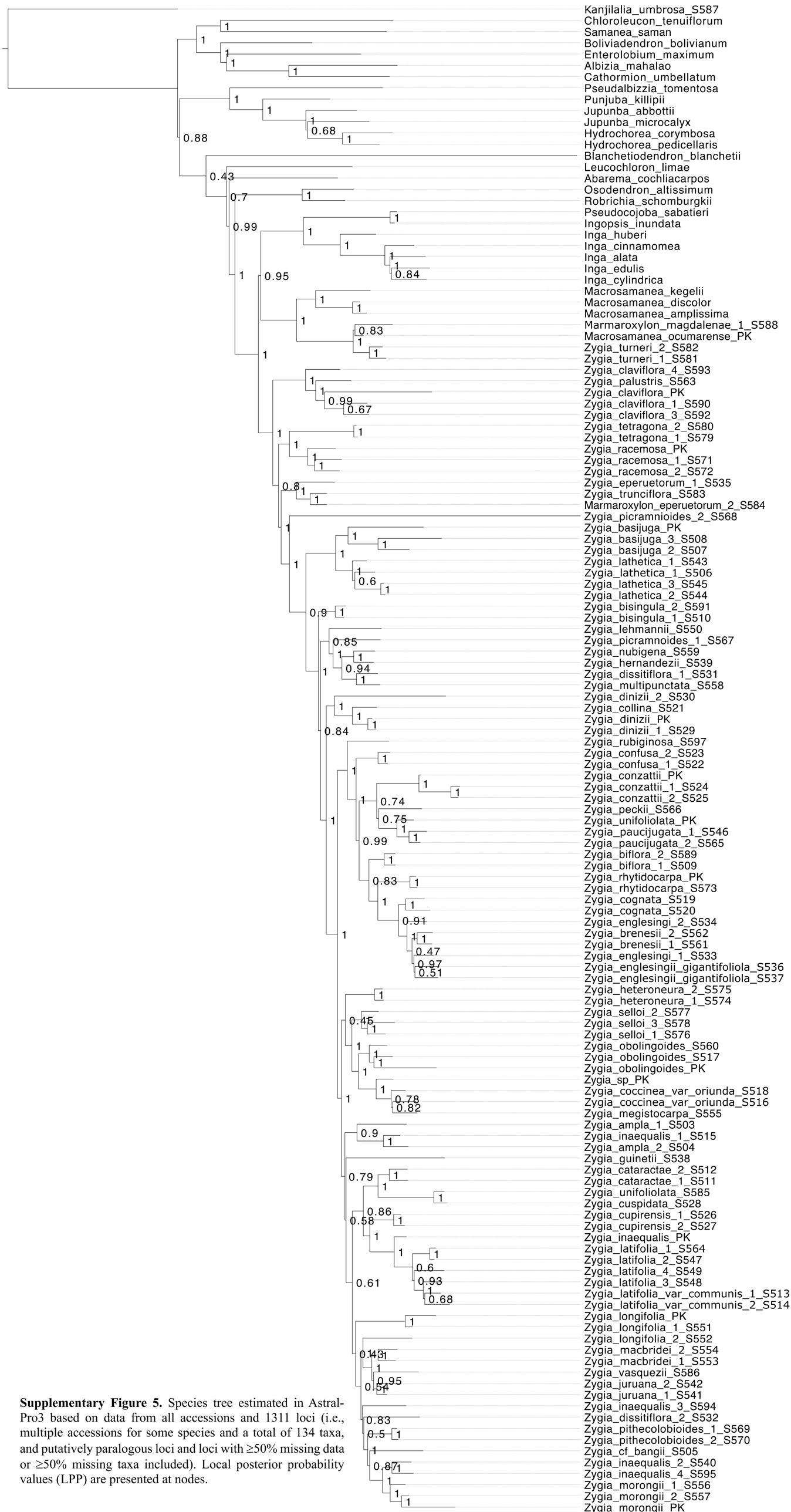

Supplement: Supplementary file 1 [file SupplementaryFile1.zip › Supplementary_Fig.5_with_caption_Ferm_et_al_2026.pdf]

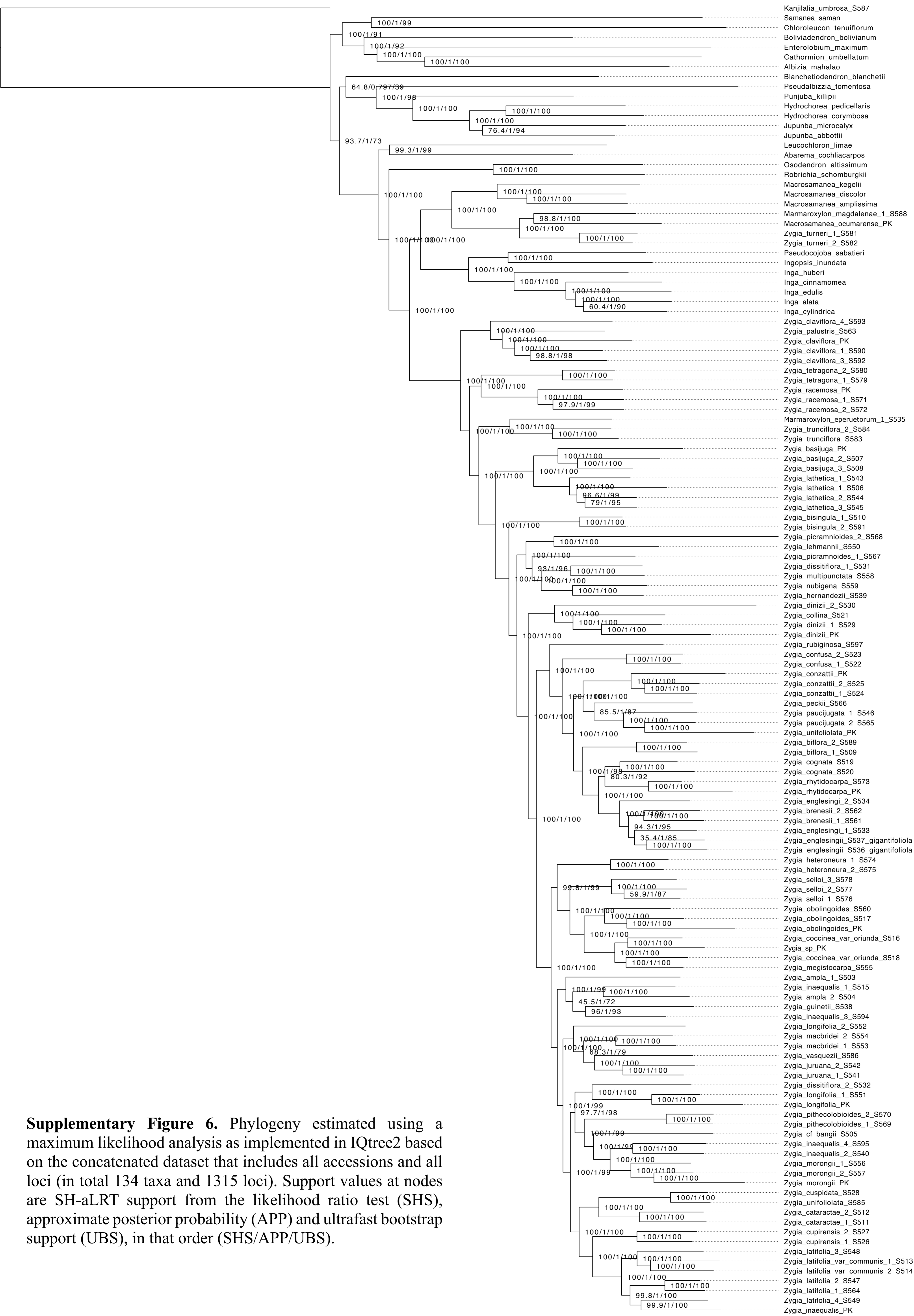

Supplement: Supplementary file 1 [file SupplementaryFile1.zip › Supplementary_Fig.6_with_caption_Ferm_et_al_2026.pdf]
