# Supplementary material for: Improved phylogenetic resolution within the Neotropical rainforest genus Zygia (Mimoseae, Fabaceae) using phylogenomic data
Source: Front Plant Sci. 2026 Jun 12;17:1816329. doi: 10.3389/fpls.2026.1816329 (PMC13303398; doi:10.3389/fpls.2026.1816329)
Supplement: Supplementary file 2 [file SupplementaryFile2.zip › Supplementary_Table2_RF_values_Ferm_et_al_2026.docx]

**Supplementary Table 2.** Robinson-Foulds statistics of the phylogenetic tree comparisons made in this study.

| **Trees/data sets** | **Raw Robinson-Foulds** | **Normalized Robinson-Foulds** |
| --- | --- | --- |
|  |  |  |
| **All samples** |  |  |
| Fig. 2^1^ vs. Supplementary Fig. 4^2^ | 18 | 0.07 |
| Fig. 2^1^ vs. Supplementary Fig. 5^2^ | 16 | 0.06 |
| Supplementary Fig. 4^2^ vs. Supplementary Fig. 5^2^ | 14 | 0.05 |
|  |  |  |
| **One sample** |  |  |
| Supplementary Fig. 1^1^ vs. Supplementary Fig. 2^2^ | 6 | 0.04 |
| Supplementary Fig. 1^1^ vs. Supplementary Fig. 3^2^ | 8 | 0.05 |
| Supplementary Fig. 2^2^ vs. Supplementary Fig. 3^2^ | 2 | 0.01 |
|  |  |  |
| **Concatenated datasets** |  |  |
| Fig. 3^1^ vs. Supplementary Fig. 6^2^ | 22 | 0.08 |
| Fig. 3^1^ vs. Fig. 2^1^ | 54 | 0.21 |
| Supplementary Fig. 6^2^ vs. Supplementary Fig. 4^2^ | 38 | 0.14 |
| Supplementary Fig. 6^2^ vs. Fig. 2^1^ | 46 | 0.17 |

^1^ Putatively paralogous loci and loci with ≥50% missing data or ≥50% missing taxa excluded

^2^ All loci included
