# Supplementary material for: Improved phylogenetic resolution within the Neotropical rainforest genus Zygia (Mimoseae, Fabaceae) using phylogenomic data
Source: Front Plant Sci. 2026 Jun 12;17:1816329. doi: 10.3389/fpls.2026.1816329 (PMC13303398; doi:10.3389/fpls.2026.1816329)
Supplement: Supplementary file 2 [file SupplementaryFile2.zip › Supplementary_Table3_Herbarium_specimens_Ferm_et_al_2026.docx]

**Supplementary Table 3.** Herbarium specimens consulted for this study. Specimens sequenced for this study are marked with an asterisk.

| **Species** | **Voucher** |
| --- | --- |
|  |  |
| **Clade A** |  |
| *Zygia turneri* | Garcia 3839 (K)*, Ramos 300 (K)*, McVaugh 20980 (Isotype MO), Sánchez 13099 (NY) |
| *Marmaroxylon magdalenae* | Haught 2097 (Holotype S)* |
| *Macrosamanea ocumarensis* | Stergios 14780 (MO)*, Pittier 14099 (Isotype K), Grimes 3153 (NY), Stergios & Velásquez 14780 (NY), Meier 2099 (NY), Steyermark 106939 (NY) |
|  |  |
| **Clade B** |  |
| *Zygia claviflora* | Ducke 35530 (NY)*, Ducke 20169 (S)*, Ducke 325 (NY), Chaviel 366 (NY)*, Yanez 151 (NY), Velazco 1703 (NY), Velazco 1129 (NY), Silva 4442 (NY), Nascimento 71 (NY), Guánchez 914 (NY), Guánchez 3604 (NY), |
| *Zygia palustris* | Thomas 3394 (NY)*, Thomas 3393 (NY), Cowan 2013 (NY), Maguire 41958 (Holotype NY), Maguire 36724 (NY), Liesner 16995 (NY), Gentry 47265 (NY), Gentry 47257 (NY), Redden 3516 (NY) |
| **Clade D** |  |
| *Marmaroxylon eperuetorum* | Fanshawe 1434 (Holotype K; Isotype NY), s. coll. 3563 (K)*, s. coll. 4170 c.f. 3563 (K) |
| *Zygia trunciflora* | Ducke 16786 (MO), Freitas 668 (K; NY)*,  Oliveira 171 (K)*, Nascimento INPA/WWF 1105.250 (NY), Grimes 3124 (NY), Ducke 809 (NY), Ackerly WWF 1105.250.2 (NY) |
|  |  |
