# Supplementary material for: Improved phylogenetic resolution within the Neotropical rainforest genus Zygia (Mimoseae, Fabaceae) using phylogenomic data
Source: Front Plant Sci. 2026 Jun 12;17:1816329. doi: 10.3389/fpls.2026.1816329 (PMC13303398; doi:10.3389/fpls.2026.1816329)
Supplement: Supplementary file 1 [file Supplementary_Fig.4_with_caption_Ferm_et_al_2026.pdf › Supplementary_Fig.4_with_caption_Ferm_et_al_2026.pdf]

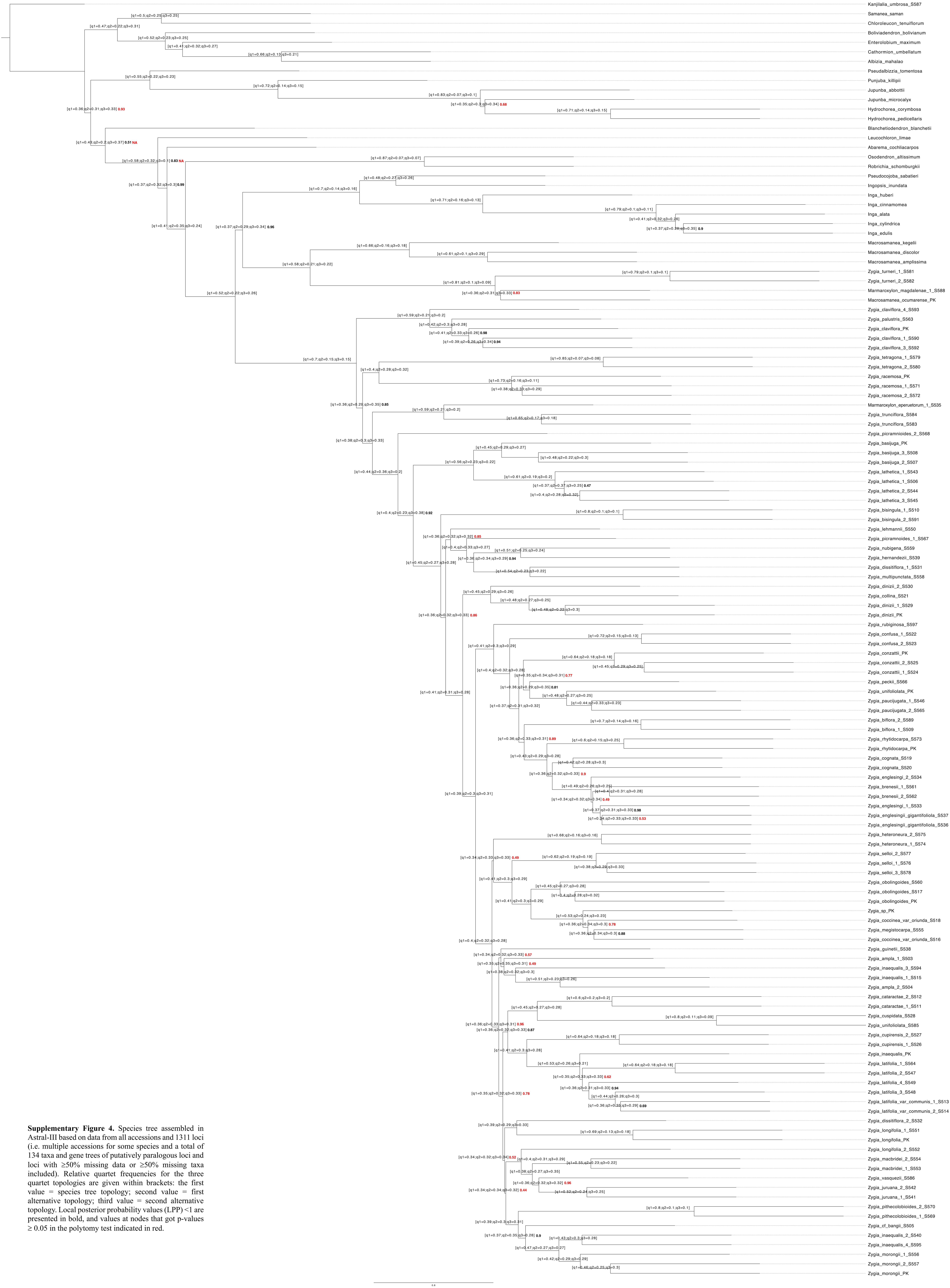

Supplementary Figure 4. Species tree assembled in Astral-III based on data from all accessions and 1311 loci (i.e. multiple accessions for some species and a total of 134 taxa and gene trees of putatively paralogous loci and loci with ≥50% missing data or ≥50% missing taxa included). Relative quartet frequencies for the three quartet topologies are given within brackets: the first value = species tree topology; second value = first alternative topology; third value = second alternative topology. Local posterior probability values (LPP) <1 are presented in bold, and values at nodes that got p-values ≥ 0.05 in the polytomy test indicated in red.
